# A Case of Cast Nephropathy Found as the Cause of Severe Renal Failure

**DOI:** 10.7759/cureus.19135

**Published:** 2021-10-29

**Authors:** Gen Adachi, Naoto Mouri, Ryuichi Ohta, Chiak Sano

**Affiliations:** 1 Community Medicine Management, Shimane University Faculty of Medicine, Izumo, JPN; 2 Community Care, Unnan City Hospital, Unnan, JPN

**Keywords:** geriatrics, renal biopsy, renal failure, multiple myeloma, cast nephropathy

## Abstract

Cast nephropathy is rare kidney disease with slow progression. It is associated with multiple myeloma (MM). In this study, we report a case of cast nephropathy in an 86-year-old woman who was previously independent in her activities of daily living (ADLs). However, she was found to have severe renal impairment after vomiting and a decrease in her ADLs. Blood and urine tests revealed the renal disorder. The patient was immediately treated with hemodiafiltration. IgG, IgA, and IgM levels were decreased by immunoelectrophoresis. A kidney biopsy showed crystals and periodic acid-Schiff stain (PAS)-negative urinary casts in the tubules. Bence Jones protein-lambda type M protein was detected in the urine. The patient was diagnosed with cast nephropathy due to MM. Hemodialysis was continued. The patient's family and the patient did not wish to initiate chemotherapy. The decision was made to follow the patient's progress. In this case, a patient who was originally independent in her ADLs developed severe renal failure with an acute course. This case suggests the importance of differentiating MM based on renal failure, even if the presentation is not typical, as elderly patients may have an atypical presentation of the disease, which can avoid invasive procedures such as renal biopsy.

## Introduction

Cast nephropathy is a rare renal disorder caused by tubulointerstitial lesions in patients with multiple myeloma (MM). It is caused by an excessive increase in monoclonal immunoglobulin light chains. These flow into Henle's loop, bind to the Tamm-Horsfall protein, and form urinary casts that obstruct the ascending limb of Henle's loop and the distal tubule. As a result, the pressure in the renal tubules (Bowman's capsule) increases, resulting in a decrease in the glomerular filtration rate (GFR) and a decline in renal function [1]. Progression from asymptomatic myeloma to symptomatic myeloma or systemic amyloidosis occurs at a rate of 10% per year for the first five years after diagnosis, 3% per year for the next five years, and 1% per year after 10 years (median of approximately 2 years) [2]. In addition, overall survival correlates significantly with the highest eGFR, with a hazard ratio of 1.6 for every 15 ml/min/1.73 m^2^ of estimated GFR (eGFR) below 45 ml/min/1.73 m^2^, as shown by a retrospective study [3]. In general, the course of cast nephropathy is considered to be gradual. The diagnosis of MM or primary amyloidosis often precedes the onset of renal failure, followed by progression to end-stage renal failure. However, it has been emphasized that crystalline casts of immunoglobulin light chains are rarely observed and can cause acute exacerbation. In this study, we encountered a case of cast nephropathy in which crystalline casts were observed. The patient was found to have a severe renal disorder at the time of admission. Not only was this a rare presentation, but it was also a case of acute renal failure in an 86-year-old patient. This condition necessitated a discussion regarding the extent of scrutiny going forward. Therefore, we report this case, as it may suggest the importance of considering cast nephropathy caused by MM as one of the differential diagnoses of advanced renal failure among older patients.

## Case presentation

An 86-year-old woman was admitted to our hospital with a chief complaint of fatigue and acute renal failure. She was scheduled to be admitted to the orthopedic department for pain control of lumbar spinal canal stenosis. However, she vomited once in the morning a week before presentation and was unable to bathe without assistance two days prior to admission. Blood tests revealed severe renal impairment. The patient was referred to the department of internal medicine for consultation on the day of admission. This patient’s medical history included medial osteoarthritis of the left knee, lumbar spinal canal stenosis, and an L2 vertebral fracture two years before admission. There were no malignant findings. She was followed by her general practitioner for blood tests every six months. Her creatinine was 0.64 mg/dL eight months prior to the hospital visit and 1.14 mg/dL one month prior to the hospital admission with no further investigation. Her vital statistics were as follows: height 142 cm, weight 41.8 kg; blood pressure 145/76 mmHg, pulse rate 74 beats/min, body temperature 36.4°C, respiratory rate 16 breaths/min, and oxygen saturation (SpO2) 98%. Blood tests revealed severe renal dysfunction with the following levels: blood urea nitrogen 139.9 mg/dL, creatinine 16.81 mg/dL, Na 134 mEq/l, K 7.5 mEq/l, Cl 100 mEq/l, Ca 8.1 mg/dL, P 11.4 mg/dL, and erythrocyte sedimentation rate (ESR) 101 mm/hour. Urinalysis showed white blood cells (2+), protein (2+), occult blood (1+), and granular and waxy casts in the urine sediments on the fourth day of admission (Tables 1-2). Blood gas tests revealed the following: pH 7.146, partial pressure of carbon dioxide (PCO2) 21.6 mmHg, bicarbonate (HCO3) 7.2 mmol/L, cK+ 7.5 mmol/L, cLac 0.4 mmol/L, and AnGap 16.9 mmol/L. The lab tests indicated metabolic acidosis with an enlarged anion gap.

**Table 1:** Laboratory values of the patient (Day 1)

Marker	Level	Reference
White blood cell	8.7	3.5-9.1 x10^3/μ
Red blood cell	3.03	3.76-5.50 x10^6/μ
Hemoglobin	9.5	11.3-15.2 g/dL
Hematocrit	28.7	33.4-44.9
MCV (Mean corpuscular volume)	94.9	79.0-100.0 fl
MCH (Mean corpuscular hemoglobin)	31.4	26.3-34.3 pg
MCHC (Mean corpuscular hemoglobin concentration)	33.1	30.7-36.6 g/dL
RDW (Red blood cell distribution width)	13.1	0.2-1.2 ％
Platelet	10.9	13.0-36.9 x10^4/μ
Neutrophil	90.2	44.0-72.0 ％
Lymphocyte	6/8	18.0-59.0 ％
Monocyte	2.6	0.0-12.0 ％
Eosinophil	0	0.0-10.0 ％
Basophil	0.4	0.0-3.0 ％
Total bilirubin	0.3	0.2-1.2 mg/dL
Direct bilirubin	0.1	<0.3 mg/dL
AST (Aspartate aminotransferase)	10	8-38 IU/l
ALT (Alanine aminotransferase)	15	4-43 IU/l
ALP (Alkaline phosphatase)	107	106-322 U/L
LAP (leucine aminopeptidase)	55	80-160 IU/l
γ-GTP (γ-glutamyl transpeptidase)	26	<48 IU/l
Cholinesterase	278	201-421 IU/l
LDH (Lactate dehydrogenase)	237	121-245 U/L
ZTT (Zinktrübungstest)	0.7	2-12 K.U
Total protein	7.5	6.5-8.3 g/dL
Albumin	3.7	3.8-5.3 g/dL
Amylase	270	44-132 IU/l
Blood sugar	81	60-109 mg/dL
Total cholesterol	166	130-219 mg/dL
Uric acid	6/6	2.3-7.0 mg/dL
BUN (Blood urea nitrogen)	139.9	8-20 mg/dL
Creatinine	16.81	0.47-0.49 mg/dL
Serum Na	134	135-150 mEq/l
Serum K	7.5	3.5-5.3 mEq/l
Serum Cl	100	98-110 mEq/l
Serum Ca	8.1	3.5-5.3 mg/dL
Serum P	11.4	0.2-1.2 mg/dL
CK (Creatine kinase)	134	41-153 U/L
CRP (C-reactive protein)	0.45	<0.3 mg/dL
eGFR (Estimated glomerular filtration rate)	1.8	<60.0 ml/min/1
Prothrombin time	136.7	70-140 ％
PT-INR (Prothrombin time-international normalized ratio)	0.86	0.80-1.20
APTT (Activated partial thromboplastin time)	25.9	23-40 sec
Treponema pallidum antibody	0.02	<0.5 S/CO
Hepatitis B surface antigen	0	<0,05 IU/mL
Hepatitis C virus antibody	0.03	<1.0 S/CO
BNP (Brain natriuretic peptide)	34.5	<18.4 pg/ml

**Table 2:** Urine test

	Day 1	Day 4
White blood cell	(2+)	(3+)
Nitrite	(-)	(-)
Protein	(2+)	(1+)
Sugar	(-)	(-)
Bilirubin	(-)	(-)
Ketone bodies	(-)	(-)
Occult blood	(1+)	(3+)
Red blood cell		> 50/HPF (high power field)
White blood cell		>50/HPF
Squamous epithelial cells		<1/HPF
Granular casts		3-9/10LPF (low power field)
Waxy casts		3-9/10LPF
Protein amount		106 mg/dL
Estimated daily protein amount		3.26 g/1.73㎡

**Figure 1:**
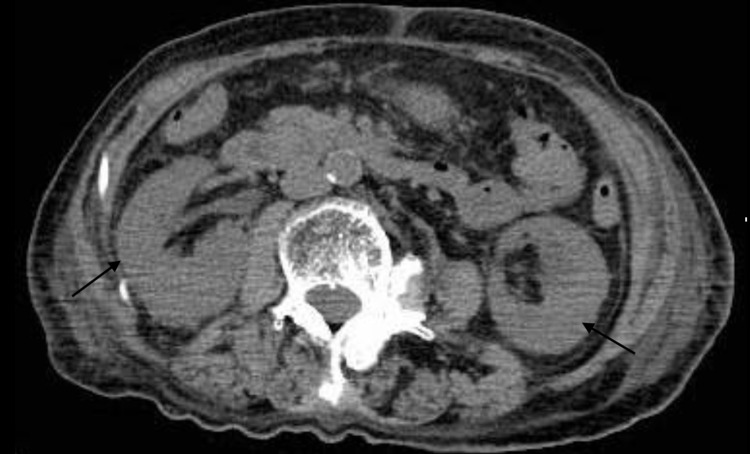
Abdominal CT There is no obvious renal atrophy.

**Figure 2:**
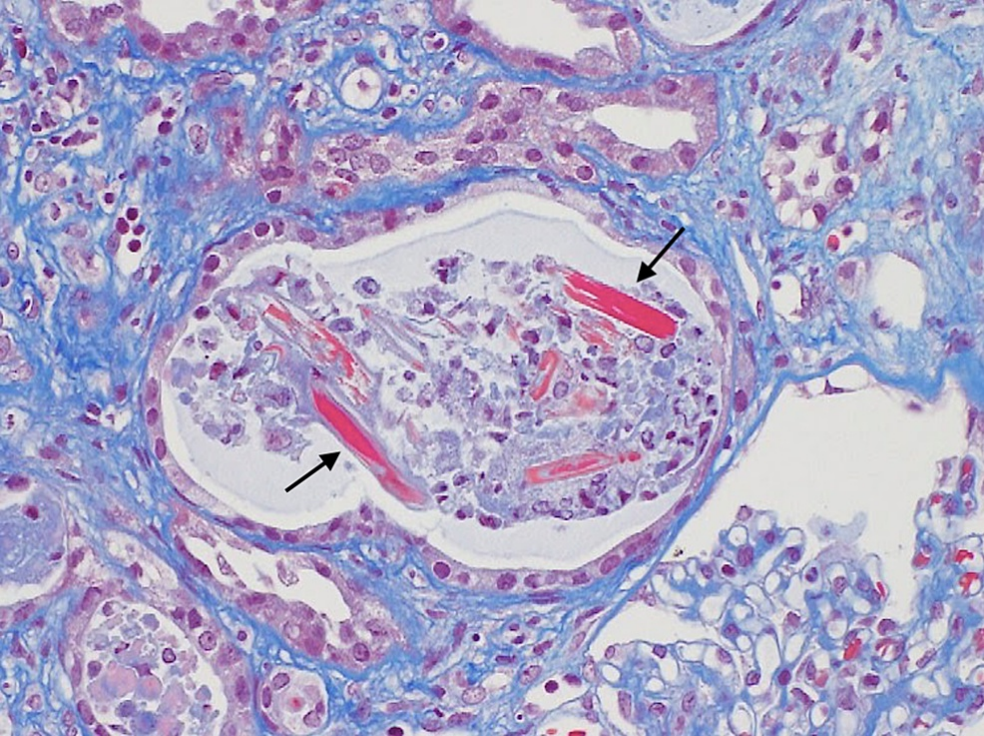
Masson staining for renal biopsy Crystalline structures with a reddish stain can be seen in tubules.

**Figure 3:**
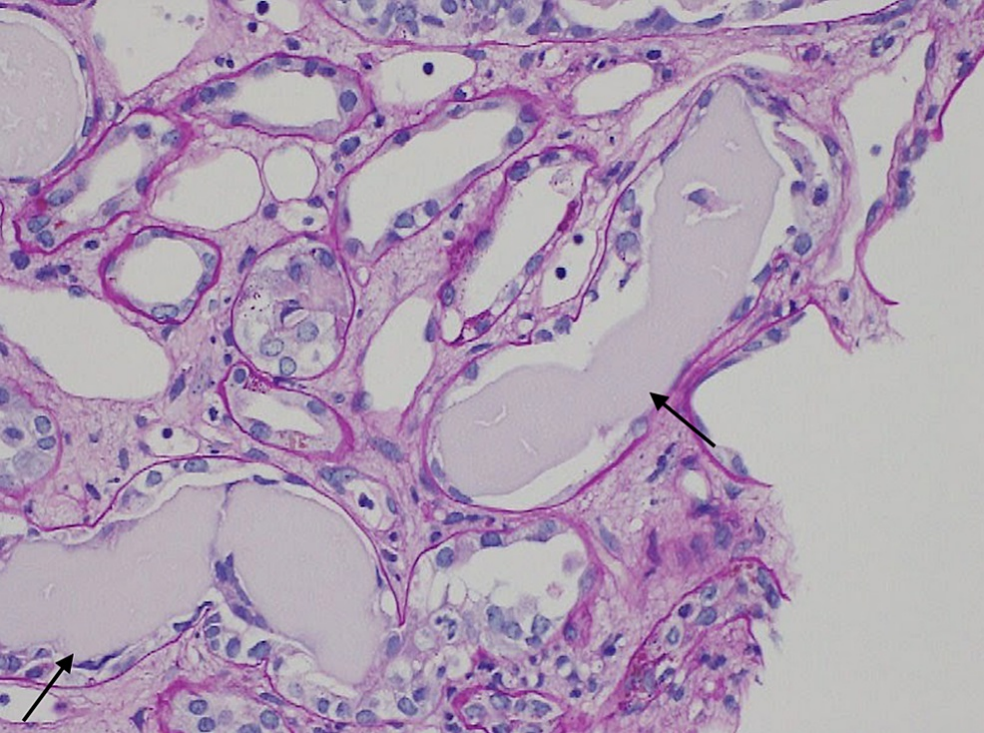
PAS stain for renal biopsy PAS-negative urinary casts can be seen. PAS: periodic acid-Schiff

**Figure 4:**
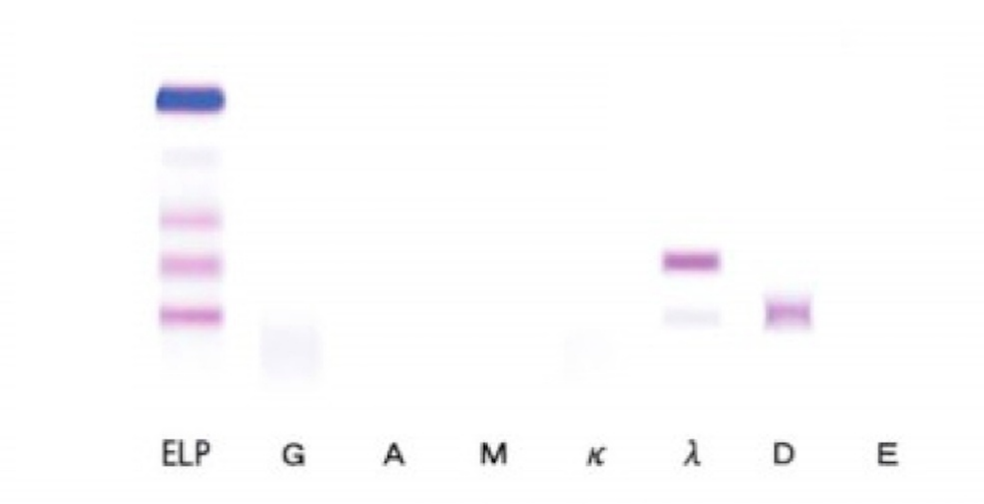
Immunoelectrophoresis (specific antiserum) ELP, electrophoresis of protein; G, anti-IgG antibody; A, anti-IgA antibody; M, anti-IgM antibody; κ, anti-Igκ antibody; λ, anti-Igλ antibody; D, anti-IgD antibody; E, anti-IgE antibody; IgD-lambda M protein is observed

**Figure 5:**
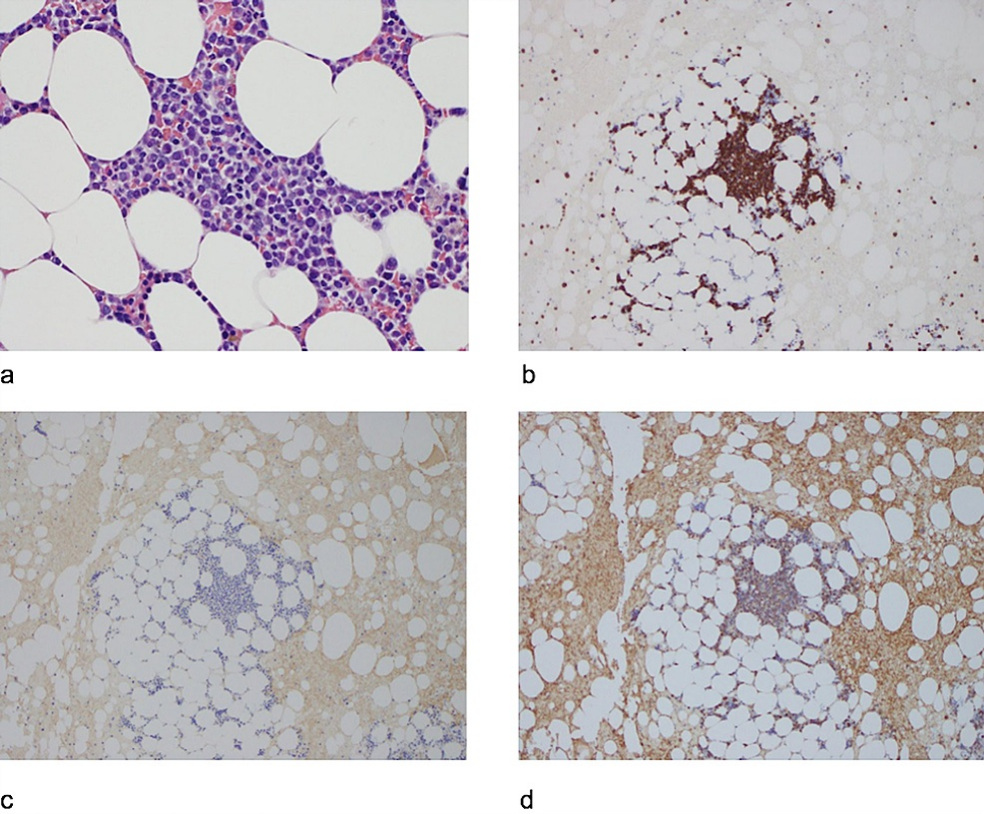
Bone marrow biopsy a. Hematoxylin-eosin stain, b. CD138 stain, c. Kappa stain, d. Lambda stain

Emergency hemodialysis with a right internal jugular vein catheter was initiated on the second day of admission. Considering further rapid progression, steroids were urgently administered on the third day of admission due to suspicion of antineutrophil cytoplasmic antibody (ANCA)-associated glomerulonephropathy. This was performed prior to a bone marrow biopsy and immunofixation electrophoresis. Considering renal atrophy and renal damage due to malignancy, an abdominal CT, head MRI, and upper gastrointestinal endoscopy were performed. However, there was no renal atrophy, and no obvious malignant findings were observed (Figure 1). Additional tests for antinuclear antibody, anti-streptolysin 0 antibody, anti-glomerular basement membrane antibody, and anticardiolipin antibody were all normal. The serum immunoelectrophoresis that we had submitted on the second day of admission came back on the eighth day, and the IgG, IgA, and IgM had decreased. On the twentieth day of admission, a renal biopsy of the right kidney was performed. Crystals in the tubules and periodic acid-Schiff (PAS)-negative urinary casts suggested acute kidney injury associated with cast nephropathy (Figures 2-3). Additional investigations were performed to determine the cause of the cast nephropathy. On the 33rd day of admission, Bence-Jones protein-λ M protein was detected in the urine. And IgD-λ M protein and Bence-Jones protein-λ protein were detected on the 36th day (Figure 4). Additional blood data showed a λ type free light chain of 8440 mg/L (κ/λ ratio of <0.001). This indicated light chain MM or monoclonal gammaglobulinemia (MGRS). Bone marrow examination showed scattered plasma cell nests of 23%, which was consistent with MM (Figure 5). Finally, the patient was diagnosed with IgD-λ type MM. There were indications for treatment. However, based on a discussion with a hematologist, it was concluded that active treatment for myeloma would not improve the patient’s condition. This patient was bedridden and her condition was poor. The side effects of the treatment could have impinged on her appetite and condition. When we discussed this with the patient, she refused to undergo chemotherapy or further active treatment. As a result of this discussion, we decided to treat her with palliative measures. For the anemia, we employed a blood transfusion and a darbepoetin alfa injection. Maintenance dialysis was initiated for the chronic renal failure. As it was difficult for the patient to return home, she was admitted to a nursing care facility.

## Discussion

Herein, we report a case of cast nephropathy caused by IgD-type MM. The cause of severe renal failure was found incidentally in an 86-year-old patient who came to the hospital with a complaint of back pain. Although cast nephropathy generally has a slow course, in this case, we considered the possibility of renal damage due to systemic disease. The patient was found to be in a severe state and was of advanced years. A renal biopsy was performed and a precise diagnosis of cast nephropathy due to MM was made. Normally, the starting point to suspecting MM is hypercalcemia and the gap between serum total protein and albumin. The flow of diagnosis for MM is to identify M-protein by serum protein fractionation and serum/urine protein electrophoresis and to confirm the diagnosis by bone marrow puncture. However, in this case, due to the atypical presentation of no hypercalcemia, high serum total protein, and low albumin, the first step was to discover cast nephropathy by renal biopsy.

A renal biopsy has been reported to reveal the diagnosis in 15-33% of patients aged 60-65 years and older [4-7]. This suggests that a renal biopsy may provide valuable diagnostic and prognostic data even in patients aged 80 years and older [8]. Dehydration, medication, nephrosclerosis, diabetic nephropathy, chronic glomerulonephritis, and ureteral stones have been suggested as probable causes of renal failure in older patients. However, when focusing on renal failure, the possibility of IgA and other immune-mediated glomerulonephritis, as well as associated nephrotic syndrome and vasculitis, makes a diagnosis by renal biopsy essential [9]. Physiological atrophy of the kidneys and shrinkage of the renal parenchyma due to aging makes renal biopsy difficult. It has been reported that complications from a renal biopsy performed with appropriate techniques are not more common in older patients than in younger patients [10]. It should be considered in Japan, where older patients are increasingly being treated in clinical practice [11-12]. In a questionnaire survey of 168 facilities in Japan, 57% of the respondents were between 80 and 89 years of age, and 14% were 90 years of age or older. This is the maximum age to consider a kidney biopsy. In other words, approximately 30% of hospitals do not consider a kidney biopsy for patients in their 80s or older in Japan [13]. Ageism should not prevent renal biopsy when it is useful, and it can be helpful in this case for diagnosis. However, it is undeniable that renal biopsy could have been avoided if MM had been listed as the differential diagnosis in this case.

In our case, the dissociation of total protein and albumin was not prominent, serum calcium was not elevated, and the patient was older and acutely ill. These atypical clinical presentations made it difficult to differentiate the diagnoses. One reason for this may be that the patient had an IgD-type MM. In our hospital, IgD is not routinely measured by serum immunoelectrophoresis, so we missed it. IgD MM is very rare, accounting for only 2% of all MM cases and clinical symptoms are often uncommon. Of the IgD MM cases, 82% had renal impairment, 37% had hypercalcemia, and the total serum protein averages were 6.8 g/dL [14]. As aging societies progress, the number of older patients with various complications is likely to increase. In this case, the patient originally had osteoporosis and was receiving bisphosphonates. This may have masked the hypercalcemia that should have occurred in MM. In this context, we should consider the ADLs and the possibility of treatment even in persons of advanced years. We should provide appropriate scrutiny and treatment for renal disorders without falling into the category of ageism. Considering the possibility of inadequate investigation of chronic renal failure in older persons due to ageism, clinicians should provide the appropriate diagnosis and treatments for these patients.

The pathology behind cast nephropathy involves immunoglobulin light chains, which are originally mostly reabsorbed by the proximal tubules. These are produced in large quantities, as in MM, and exceed the capacity of the proximal tubules to process them. In the distal tubules and collecting ducts in the kidneys, these particles aggregate to form cylindrical casts. There are two hypotheses regarding the mechanism of the acute onset of severe renal failure in this case. First, there may have been an event in the background that significantly reduced the reabsorption capacity of immunoglobulin light chains in the proximal tubules. Second, the production of immunoglobulin light chains increased rapidly and progressed gradually. However, it suddenly manifested when the threshold was exceeded. These pathological conditions were also observed in the present case. These conditions may have occurred simultaneously. Factors that promote cast formation include dehydration, infection, hypercalcemia, contrast media, non-steroidal anti-inflammatory drugs (NSAIDs), and diuretics. Although there were no findings of dehydration or infection, in this case, the use of celecoxib was a suspected drug. This may have had a significant impact on renal damage in this case. NSAIDs suppress vasodilation by inhibiting the production of prostaglandins, resulting in pre-renal damage due to decreased renal blood flow. Although there is less risk of gastrointestinal damage and bleeding with COX-2 selective inhibitors, renal damage is thought to occur in the same manner as with nonselective NSAIDs. Caution is required with the use of these agents [15]. There are also data showing that 20-30% of patients with MM have renal failure at diagnosis, 3-9% of newly diagnosed patients with MM will have severe renal impairment requiring dialysis, and 70% of patients requiring dialysis will have cast nephropathy. It is estimated that 70% of patients requiring dialysis have cast nephropathy [16]. Therefore, it is estimated that a small percentage of patients develop cast nephropathy as acute-onset kidney injury. The mechanism is thought to be a rapid increase in immunoglobulin short-chain production due to alterations in MM disease [16]. In our case, the creatinine level gradually worsened in one year. The fact that the disease progressed gradually and worsened rapidly within a month suggests that these pathological conditions were in place. It is necessary for clinicians to always consider the diseases behind the rapid deterioration of renal function in older patients. They must be aware of the exacerbation of the disease and the possibility that drug use may further aggravate the condition.

## Conclusions

It is important to differentiate cast nephropathy from advanced renal failure among older adults, even if it is an acute clinical course. Rapid progression of cast nephropathy can be caused by the accumulation of short immunoglobulin chains through various predisposing factors, as in this case. In particular, older patients tend to have multiple comorbidities and take various medications. This can complicate the pathophysiology and make the clinical presentation uncommon. In this situation, a pathological biopsy is important as a decisive factor in the differential diagnosis. The indications for biopsy should be carefully considered, even in persons of advanced years. Appropriate scrutiny with MM in the differential diagnosis can avoid unnecessary renal biopsy. Older patients with acutely advanced renal failure should be investigated considering the possibility of MM, even if the presentation is atypical.
